# The Mechanism of Moxibustion: Ancient Theory and Modern Research

**DOI:** 10.1155/2013/379291

**Published:** 2013-09-12

**Authors:** Hongyong Deng, Xueyong Shen

**Affiliations:** ^1^Shanghai University of Traditional Chinese Medicine, Shanghai 201203, China; ^2^Shanghai Research Center of Acupuncture and Meridians, Shanghai 201203, China

## Abstract

The moxibustion has a dual effect of tonification and purgation in TCM theories, which are based on two aspects: the actions of the meridian system and the roles of moxa and fire. Modern research works of the moxibustion mechanism mainly relate to the thermal effects, radiation effects, and pharmacological actions of moxa and its combustion products. Experimental results showed that moxibustion thermal stimulation affects both shallow and deep tissues of the skin, and the warm-heat effects of moxibustion have a close relation to the warm receptors or/and the polymodal receptor. The burning moxa radiation spectrum ranges from 0.8 to 5.6 **μ**m; peak is nearby 1.5 **μ**m, lying within the near infrared portion. There is an amazing consistency in the infrared spectrums of three types of indirect moxibustion and the unified spectrum of acupoints; all have their peaks of radiation near 10 **μ**m. Lots of ingredients had been identified from mugwort leaves and moxa smoke, which have a variety of biological activities; they were considered to participate in the comprehensive effects of moxibustion. Although lots of research works have been carried out and made some progress, there is still a great distance from fully understanding the mechanism of moxibustion.

## 1. Introduction

Moxibustion is a kind of external treatment; it is based on the theory of traditional Chinese medicine (TCM), and it usually bakes acupoints with burning moxa wool. Moxibustion can dredge meridians and regulate qi-blood and has been used to prevent and cure diseases for more than 2500 years. *Zuo zhuan* of the pre-Qin dynasty in China, which recorded a disease discussion occurred in 581 B.C., is considered to be the earliest literature of moxibustion. The silk books discovered in Mawangdui tomb of the Han dynasty (about 168 B.C.), *Moxibustion Classic of Eleven Foot-hand Meridians* and *Prescriptions for Fifty-two Diseases*, had documented the use of moxibustion to treat complex diseases. There are a lot of moxibustion contents in *Inner Canon of Huangdi*; it inferred that the origin of moxibustion is related to the living habits and disease characteristics of northern Alpine nation in the part of *Su wen, Yi fa fang yi lun*. Later doctors after Han dynasty had made considerable progress in theory and practice on moxibustion and promoted moxibustion to be a mature and widely used therapy.

Moxibustion has been applied in treating a great range of diseases. A bibliometric analysis on the papers published from 1954 to 2007 in China showed that up to 364 kinds of diseases can be treated with moxibustion. The most proper indications of moxibustion therapy are malposition, diarrhea, and colitis; the common proper indications are urinary incontinence and dysmenorrhea; the next common proper indications are knee osteoarthritis, temporomandibular joint disturbance syndrome, soft tissue injury, heel pain, asthma, urinary retention, and herpes zoster [1]. Moxibustion can also be used to treat weakness, fatigue, and aging related problems. Moxibustion can be classified as traditional moxibustion, drug moxibustion, and modern moxibustion. Traditional moxibustion therapy is the most commonly used in the ancient and contemporary moxibustion clinics; it is characterized by the use of moxa as burning material and can be divided into direct moxibustion and indirect moxibustion depending on whether moxa is directly in contact with the skin while operating. A moxa cone placed directly on the skin and ignited is called direct moxibustion, while the moxa kept at certain distance from the skin is called indirect moxibustion. The insulating materials of indirect moxibustion can be air, garlic, ginger, aconite, salt, and so forth. Drug moxibustion, also named nature moxibustion, uses irritant drugs (such as cantharis, garlic, and semen sinapis) to coat the surface of acupoints and make local skin flushed and blistered to cure diseases. Modern moxibustions, such as microwave moxibustion, laser moxibustion, and electrothermal moxibustion, are used to simulate traditional moxibustion stimulation factors by physical or chemical methods to achieve therapeutic effects of moxibustion. Usually, narrow sense of moxibustion refers to the traditional moxibustion with moxa. This review will concentrate on the ancient theory and modern mechanism research of traditional moxibustion.

## 2. Traditional Moxibustion Theory

*Ling Shu, Guan Neng* says that where needle does not work, moxibustion does. TCM theory holds that moxibustion has a dual effect of tonification and purgation. Different from needles and drugs, characteristics of moxibustion in materials and using fire determine that its efficacy is inclined to warming and nourishing. So, moxibustion is often applied in deficiency-cold syndrome, though some excess-heat syndrome can also use it. The roles of moxibustion can be broadly grouped into warm nourishing, warm dredging, and warm melting. Warm nourishing refers to the benefits of warming Yang, tonifying qi, nurturing blood, and relieving depletion; warm dredging refers to the functions of activating blood, dissolving stasis, promoting qi, dredging channels, and relieving pain; warm melting refers to the roles of reducing phlegm, eliminating stagnation, removing wind, dispelling dampness, drawing out poison, and purging heat. Some people believe that warm dredging is the nature of moxibustion and is the key role of moxibustion effects. The functions of moxibustion, expelling cold, promoting the circulation in meridians and collaterals, clearing away heat, detoxification, and so forth, are dependant on the efficacy of moxibustion for circulating qi and blood flow [2]. 

In TCM basic theory, moxibustion effects are based on two aspects: the action of the meridian system and the role of moxa and fire.

### 2.1. Meridian System

TCM usually takes “needling” and “moxibustion” collectively, for both of them are similar therapeutics based on the same theory of meridian and acupoint. In other words, the moxibustion therapeutic effect is partly dependant on the body's nonspecific system of meridians. 

Moxibustion is closely related to meridians, cutaneous regions, and acupoints. Meridian system consists of channels and collaterals; they are pathways of communicating internal and external, contacting organs, running qi-blood, and regulating the whole body. *Ling Shu, Hai Lun* says that there are twelve regular channels, the inner ones belong to viscera and the outer ones connect with limbs. TCM believes that a person is as a whole. The organs and limbs communicate and interact through the meridian system, which plays a very important role in physiological functions and pathological processes. The cutaneous regions are the surface part of the twelve regular channels, which are nourished by channel-qi. The cutaneous regions can show the status of qi-blood from meridians and organs, also it can receive treatment stimulation and then make effects. Acupoints are the sites on the body surface, in which the qi of organs and meridians assembled, that act as target points and response points of treatment. 

In the moxibustion treatment process, the cutaneous regions and acupoints are the terminals of the meridian system, as the receivers, by which moxibustion stimulations can be transmitted into the body. Through the meridian system, moxibustion can reinforce insufficiency and reduce excessiveness and directly correct the disease state of the human body or activate the meridian system self-healing function and play a therapeutic role. For example, the different acupoints can cure different diseases in moxibustion, and the same acupoints can get similar results regardless of acupuncture or moxibustion; all of these proved that the body meridian and acupoint system play an important role in the treatment of moxibustion.

### 2.2. Moxa and Fire

*Elementary Medicine* believes that the diseases that cannot be cured by drugs and acupuncture should be treated with moxibustion. The unique therapeutic effects of moxibustion are closely related to the specificity of moxa and fire. 

On moxibustion fire in TCM, there is a discussion in *Shen jiu jing lun* stating that moxibustion using fire, for being hot and rapid, with soft body can bear with that to eliminate the shadow; it can move instead of stay and always go into organs. Fire is hot, so it can warm back the Yang and eliminate cold of the Yin, even it can melt the poisoning things caused by damp, wind, phlegm, and so on; fire is speedy, so it can dredge the channels, remove the pain or numbness, and active blood and qi. So, the feature of moxa fire shows the main role of moxibustion.

Materials are very important to moxibustion. The choosing of materials of moxibustion in TCM is really harsh.* Pu ji fang, Acupuncture* cited the *Xiao pin fang* on eight kinds of fire: moxibustion with pine wood fire, hard to cure; cedar wood fire, ulcer and pus; orange wood fire, skin hurt; mulberry wood, muscle withered; jujube wood fire, body emaciated; bamboo fire, tendons injured, excessive lead tendons flabby; trifoliate orange wood fire, veins “collapse”; elm wood fire, bone hurt, excessive lead bone withered; none of them can be used. But moxa fire is warm without dry, and it can ascend and descend with strong penetration ability into the viscera. *Compendium of Materia Medica* had said that moxa leaf are slightly bitter and over-spicy when raw, and slightly spicy and over-bitter when processed. Moxa with the nature of pure Yang, raw moxa is warm and become hot after processing. It can take the Tai-Yang fire and get back dying Yang. It can go through three Yin, get rid of all the cold and dampness, and turn the cold into warm after taking orally. Moxibustion with moxa leaf can get into the channels and cure hundreds of diseases. Its function is great. The drug properties of moxa leaves (raw) are that they turn warmer after being processed, become moxa wool (processed), which are suitable for moxibustion, and the older the better. The ancients chose moxa as moxibustion material for it is easy to collect and more for its drug properties, and long-term clinical practices have proved that.

## 3. Mechanism Research of Moxibustion

Modern research of moxibustion started in the earlies of last century, Japanese scholars began to observe physical characteristics of moxibustion materials and the effects of moxibustion on blood pressure and intestinal peristalsis in 1912 [3, 4]. Up to this day, there have been more and more studies of effects of moxibustion on the human body or experimental animals, almost involving all major physiological systems, especially in the fields of analgesic, enhancing immunity and antiaging. At the same time, researching works on the mechanism of moxibustion also gradually developed, mainly related to the thermal effects, radiation effects, and pharmacological actions of moxa and its combustion products.

### 3.1. Thermal Effects

Burning moxa without flame can produce high temperature of about 548–890°C [5, 6]; it will give a warm feeling when it isclose to the body, so some people think that this treatment is essentially a thermal physical effect [7]. Experiment confirmed that single Zhuang (a dose unit of moxibustion) of moxa cone (2 mg) moxibustion on mice abdomen can raise the temperature to 130°C outside the skin of the point and 56°C inside the skin; the same changes of temperature were not observed in the forelimb far away from the stimulation site [8]. By using 50 mg moxa cone direct moxibustion on the skin of mice with thermocouple implanted, the temperatures of epidermal, subcutaneous, and basal layers were different; the results suggested that moxibustion thermal stimulation affects both shallow and deep tissues of the skin [9]. The maximum temperature change by indirect moxibustion was about 65°C on the skin and 45°C in the subcutaneous layer [10]. The temperature-time curve of moxa cone can be characterized by slow rising, rapid rising, rapid decline, and slow decline phases, and ginger-separated moxibustion can “buffer” the temperature changes [11]. The actual temperature of indirect moxibustion is greatly affected by the texture, size, and the moisture content of the insulating material [12].

The thermal effects of different moxibustions are not the same. Some people used thermal resistor thermometer and computer online real-time processing to measure the skin temperature at the acupoints of different moxibustions: direct moxibustion, ginger-separated moxibustion, suspension moxibustion, light moxibustion, and He-Ne laser moxibustion. All of them except He-Ne laser moxibustion had significantly changed the temperature of the acupoints through the skin to the muscularis, and each had their own rules and characteristics. The results suggested that the effects on acupoint and even the efficacy of moxibustion depend on the temperature changing of acupoint caused by moxibustion [13]. Others observed the relationship between the moxibustion effect and the intensity of thermal stimulation through the change of pain threshold. In the 40~60 minutes of moxibustion, the pain threshold rose with the operative time and increasing the burning moxa amount per unit time can significantly improve the immediate analgesic effect and lingering effects [14]. Experiment of activation of subnucleus reticularis dorsalis (SRD) neuron by variety intensities of moxibustion thermal stimulation shown that noxious thermal (44–52°C) stimulation can activate SRD neurons, which reaches a plateau when the stimulated area is increased to a certain range [15].

The warm-heat effect of moxibustion has a close relation to the warm receptors (WRs) or/and the polymodal receptor (PRs). The antipyretic and thermolytic effects of moxibustion are achieved by stimulating polymodal receptors of acupoints [16–18]. Effects of moxibustion on the skin can appear as hottness, flushing, pain, blisters, and other skin irritations and burns phenomena. Moxibustion can lead to vasoconstriction at the burning point while vasodilatation around the point and increase peripheral arterial blood flow and microvascular permeability [8, 19]. Another thermal effect of moxibustion is to induce heat shock proteins (HSPs) in local tissues. HSPs are a class of functionally related proteins involved in the folding and unfolding of other proteins. As an endogenous protective mechanism, HSPs can be synthesized in cells in response to hyperthermia and other environmental stresses. The HSPs induced by moxibustion may be an important factor of its mechanism of action [20]. 

### 3.2. Radiation Effects

By irradiating acupoints of pain model rats with radiogenic heat of 40–43°C, there are no significant changes in the tail-flick latency or vocalization threshold, suggesting that not any thermal stimulation can achieve moxibustion efficacy [21]. The burning moxa emits visible light and infrared (IR) radiation; therefore, besides the heat effects, nonthermal radiation effect may be an important role in the efficacy of moxibustion. Physics tells us that the radiation is a process of energy outward diffusion in the form of electromagnetic waves or particles; any object above absolute zero in temperature emits electromagnetic radiation. At present, the common view is that the ignited moxa radiation spectrum ranges from 0.8 to 5.6 *μ*m; peak is nearby 1.5 *μ*m, lying within the near infrared (NIR) portion [22]. But results are reported differently due to the measurement methods and the experimental conditions. Thermal radiation of burning moxa stick measured by indirect methods is mainly far infrared (FIR) near NIR, with spectrum peak at 2.8 *μ*m [23]. Measured with visible-infrared monochromator, radiation spectrum of drug moxa sticks is distributed from red light through NIR to middle infrared (MIR), in which multipeaks especially at 2.4 *μ*m are detected and without the parts of wavelength shorter than 0.6 *μ*m [24].

**Figure 1:**
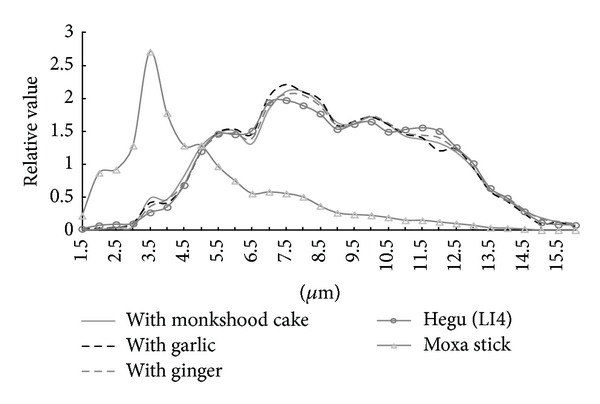
Unified infrared radiation spectrums of an acupuncture point, Hegu (LI 4), direct moxibustion with a traditional moxibustion stick, and indirect moxibustion with three traditional media.

By analyzing and comparing the infrared radiation spectrums of the moxibustion, the substitute moxibustion, and acupoints of human body, it was found that there was a surprising consistency in the spectrums of three types of indirect moxibustion, namely, separated with prepared monkshood, ginger, and garlic, and the unified spectrum of acupoints. Both had their peaks of radiation near 7.5 *μ*m (after modification, this wavelength should be around 10 *μ*m). However, the spectrum of the substitute moxibustion (separated with cucumber and carrot) was completely different from them. Its warming function was far less than the traditional moxibustion, and there was also a big difference between the infrared radiation spectrums of the moxa-stick (with a peak at 3.5 *μ*m) and acupoints (see also Figure 1 and Table 1). The results indicated that, in the therapeutic effect of traditional indirect moxibustion, the resonance vibrations of infrared radiation of indirect moxibustion and acupoints play an important role and the substitute moxibustion could not replace the traditional moxibustion in terms of the infrared characteristics of moxibustion [25–29]. 

Infrared acting on the body will produce thermal and nonthermal effects. Thermal effects are produced under the action of electromagnetic waves; the human body molecules absorb energy from IR and convert it into heat and therefore promote blood circulation and improve the cell and enzyme activities. The nonthermal effect is related to the interaction of electromagnetic waves and organism; it is more complex and with nonlinear characteristics. The actions of NIR and FIR on organism are different. NIR is generally believed to play a major role in the biological radiation effect of moxibustion. When NIR irradiates body, the light reflected by the skin is relatively low, the energy can be transmitted about 10 mm deep into the skin, reach the tissues, and be absorbed by them [30]. The NIR can induce some active substances produced within the tissues, after being absorbed by connective tissue, blood vessels, lymphatic vessels, and nerves under the irradiated skin, distribute to other parts of the body with the blood circulation, and enhance the metabolism and thermogenesis of organs they reached. NIR can also energize the metabolism of cells. The energy generated by the photoelectric effect and photochemical process and passed through the nerve-humoral system can provide the activation for the pathological cells lacking energy and then further adjust the body's immune and neurological functions [31, 32].

### 3.3. Pharmacological Actions

Moxa, *Artemisia argyi* Levl.et Vant., also known as mugwort, is a Compositae *Artemisia* perennial herb. Mugwort leaf can produce moxa wool after drying and grinding, which is a common moxibustion material. The ingredients of moxa are complicated; more than 60 kinds of components had been identified [33]. The volatile oils of moxa include 1,8-Cineole, alkenes (alpha-thujene, pinene, sabinene, etc.), camphor, borneol, and little aldehydes, ketones, phenols, alkanes, and benzene series compounds. Heptatriacontane (C_37_H_76_) plays an important role in combustion [34]. The moxa also has tannins, flavonoids, sterols, polysaccharides, trace elements, and other ingredients.

The ingredients of moxa always change according to the place and season of production. The oil rate of QiAi in Hubei is obviously higher than in Hebei, Shangdong, and other places. Some people had measured the heat of combustion from different kinds of moxa: QiAi (from Hubei) was 18139 J/g, BeiAi was 17463.4 J/g, QiAi (from Hebei) was 17419.3 J/g, and ChuanAi was 16136.4 J/g [35]. The combustion heat of QiAi (from Hubei) was the biggest, and it has been considered to be the best moxibustion material since ancient times. 

The volatile oil rate of moxa is 0.45%–1.00%. It has a variety of biological activities such as the expansion of airway smooth muscle, relieving cough, expectorant effect, and a strong antioxidant activity [36–38]. The moxa is rich in flavonoids and polysaccharides, which have strong antioxidant activity too [39, 40].

The moxa combustion test showed that the relative equilibrium moisture content of moxa was 13.51%, the relative ash content was 11.77%, and the relative smoke production rate was 126.42% [41]. Parts of the moxa combustion products are brown tar-like substances; they play a role by penetrating into the human body through the skin damaged by the burning. The moxa and the combustion products of moxa having been extracted with methanol, both extracts showed the actions of clearing the free radicals and lipid peroxidation, and the latter was stronger. The result indicated that the active ingredients of moxa were increased rather than being destroyed after burning. The methanol extracts of moxa combustion products, tars, can be divided with silica gel column chromatography, and the antioxdant components were found in band IV. Further divided by thin-layer chromatography, the antioxidant effect in band Rf 0.14 is better than the synthetic antioxidant BHT. Ginger and garlic, the important auxiliary materials for moxibustion, are commonly used in indirect moxibustion. The ginger and garlic had been put on the evaporating dish for experiment and had confirmed that gingerol and allicin, the active ingredients of them, could act on the body by heat to give the therapeutic effects [42–44]. The extracts of moxa combustion ashes also have the strong ability of antifree radical [45].

Another combustion product of moxa is smoke. The smoke of moxa contains a variety of complex components, and its volatile ingredients are ammonia, alcohols (ethylene glycol, pentyl butanol), aliphatic hydrocarbons, aromatic hydrocarbons, terpene compounds and their oxides, and so forth. They may come from the incomplete combustion products of moxa volatile oil of moxa and its oxidation products. Qualitative analysis of the smoke of burning moxa by solid phase microextraction-gas chromatography-mass spectrometry (SPME-GC-MS) had isolated 61 peaks and identified 26 ingredients. The founded substances can be divided into 3 parts by time: the furan structure substances in 0–10 min, mainly aromatic compound in 10–40 min, and esters, alkanes, or hydroxyl-containing compounds in 40–70 min [46]. The smoke of moxa can be used in air disinfection and as antiviral and antifungal. It was also reported that it has applications in wound infections, vaginal itching, uterine prolapse, anal fistula, common warts, and so forth [47], and some studies showed that the smoke of moxa would make effects on the body through breathing [48].

There is still a debate on the safety of moxa smoke. Some reports showed that moxa smoke may be harmful to the human body, such as causing allergic reactions [49, 50]. The mugwort leaf contains terpenes; it may produce polycyclic aromatic carcinogens in the process of combustion, and during moxibustion, the concentration of air pollutants, such as nitrogen oxides, carbon monoxide, and particulates, is tenfold higher than the level of standard class II which was issued in the State Environmental Protection Act. They would do damage to the patients and staffs [51]. But a research giving consideration to short-term and long-term exposure showed that the volatile matter and carbon monoxide generated by the smoke of moxa under normal operating conditions did not exceed the safety level [52].

## 4. Conclusion

On the mechanism of moxibustion effects, there have been many viewpoints, such as thermal stimulation effect, non-specific autologous protein therapeutics, non-specific stress responses, and aromatherapy. The generally accepted view is that the meridian system combines with moxibustion physical and chemical effects to produce comprehensive effects. When physical and chemical factors act on the acupoint receptors, the signal enters the central nervous system through the peripheral pathways and outgos after being integrated, adjusting the nerve-endocrine-immune network and circulatory system, so as to regulate the internal environment of the body, in order to achieve the effects of preventing and curing diseases [53]. Although lots of research works have been carried out and made some progress, there is still a great distance from fully understanding the mechanism of moxibustion. Therefore, we will propose the following views on the study of mechanism of moxibustion in the future.

First, value the importance of whole, moxibustion cannot be separated from the theory of TCM. More than a simple stimulus, meridian and acupoint system of the human body is the key of efficacy of moxibustion. The studying of mechanism of moxibustion from the overall level, based on the further understanding of the meridian system or even of the TCM system, is indeed very difficult. But on the other hand, maybe the studies of moxibustion should be helpful to the understanding of acupoint, meridian, and TCM. For example, some people had reported the phenomenon of  “heat-sensitive points” [54]; it is a useful exploration of extending the study perspective from the part to the whole with moxibustion as the breakthrough point. 

Second, pay more attention to scarring moxibustion (suppurative moxibustion). Scarring moxibustion had been the favorite to ancient doctors, “where there is moxibustion sore, there is cure.” Modern clinical practice has also shown that scarring moxibustion, compared with other moxibustions, has advantage of curative effect in the treatment of some chronic refractory diseases.

Third, it is necessary to introduce more new technologies and disciplines into the mechanism research of moxibustion effect, such as bioheat transfer theory, the interdiscipline focus heat transfer phenomena in living organisms; its purpose is to reveal the rules of energy transport in the organisms by introducing the basic theory and research methods of the heat transfer into the field of biology and medicine. The application of the interdisciplinary approach will undoubtedly promote the research of moxibustion [55].

Fourth, study on the mechanism of moxibustion should be oriented to promote its clinical application. Many research achievements have already been applied in clinic, such as the applications of 650 nm–10.6 *μ*m combined laser moxibustion on knee osteoarthritis and bradycardia [56, 57] and the multifunctional moxibustion instrument which simulate the traditional moxibustion by heating artificial moxa (contains effective components of moxa) with electromagnetic-heating device [58]. There are enough reasons to believe that, with the progress of mechanism research, the new achievements will surely provide a larger space to improve the patient experience and the curative effect of moxibustion.

## Figures and Tables

**Table 1:** Intensities and peaks wavelengths of the infrared radiation of traditional moxibustion, moxibustion with controls, and Hegu (LI4).

	*n*	Intensity of radiation (mV)	Wavelength of the peak of radiation (*μ*m)
Traditional moxa stick	4	43300.41 ± 425.15	3.5
Smokeless moxa stick	4	31.15 ± 3.49^#^	7
*555 *cigarette	4	37.03 ± 3.82^#^	3.5

Indirect moxibustion with monkshood cake	4	681.87 ± 47.52^∗∗ΔΔ^	8
Indirect moxibustion with ginger	4	520.27 ± 68.22^∗Δ^	7.5
Indirect moxibustion with garlic	4	594.79 ± 44.71^∗∗ΔΔ^	7.5
Indirect moxibustion with cucumber	4	274.47 ± 19.61	5
Indirect moxibustion with carrot	4	50.53 ± 4.68	5

LI4 (Hegu)	28	20.40 ± 5.69	7.5

^#^Compared to the traditional moxa-stick, *P* = 0.000.

*Compared to indirect moxibustion with cucumber, *P* = 0.004.

**Compared to indirect moxibustion with cucumber, *P* = 0.000.

^Δ^Compared to indirect moxibustion with carrot, *P* = 0.001.

^ΔΔ^Compared to indirect moxibustion with carrot, *P* = 0.000.
